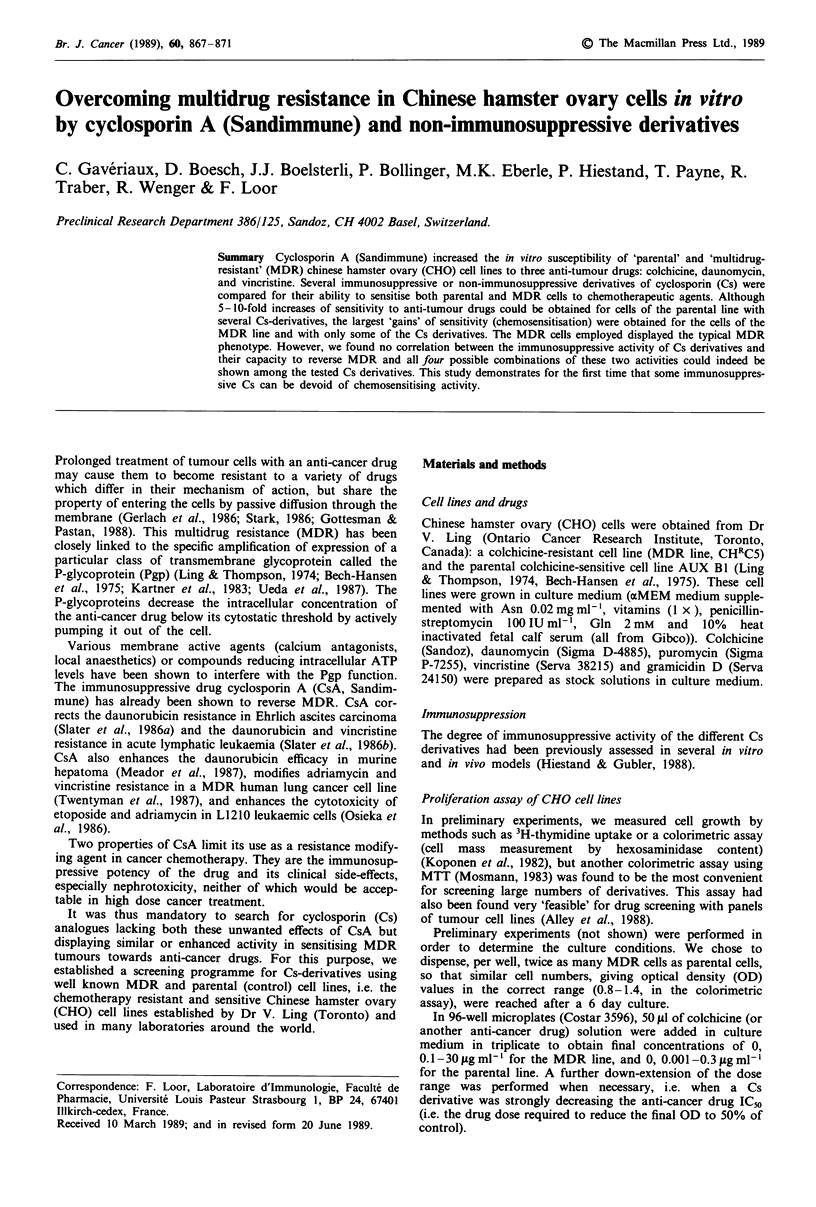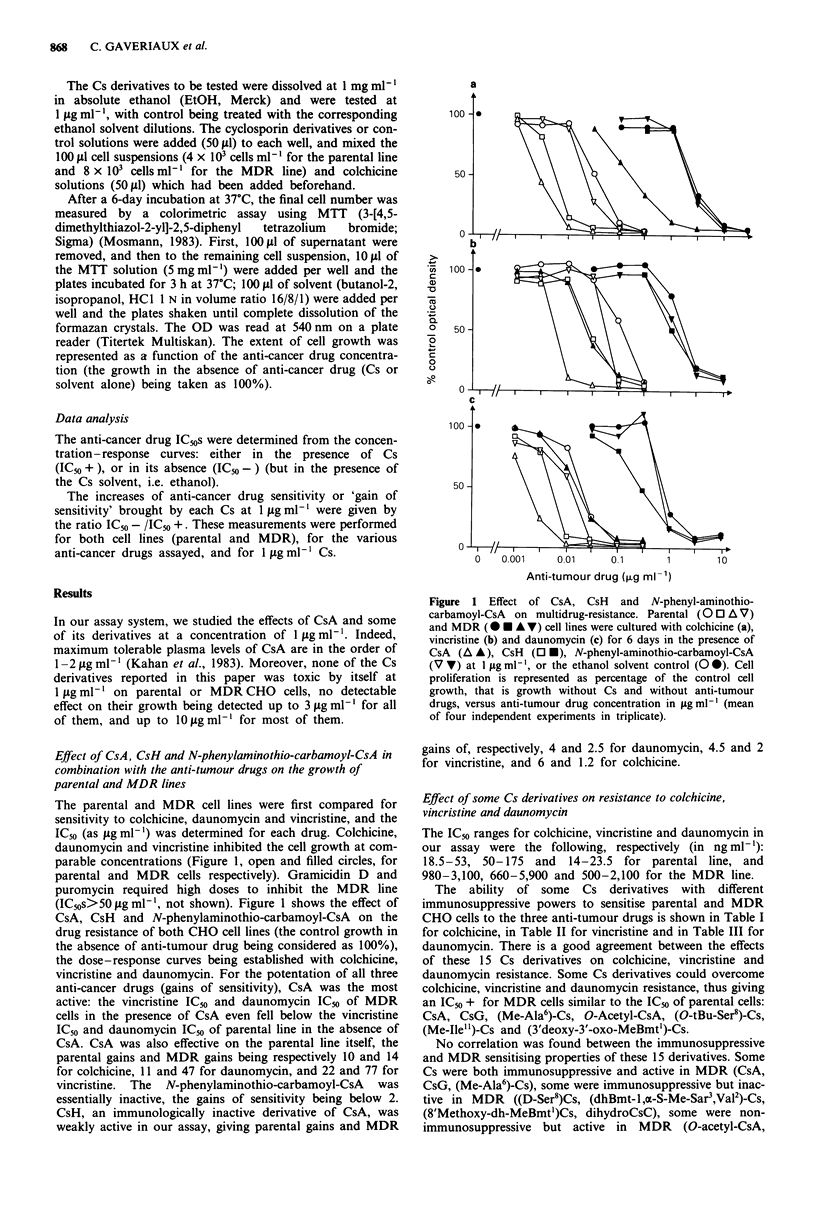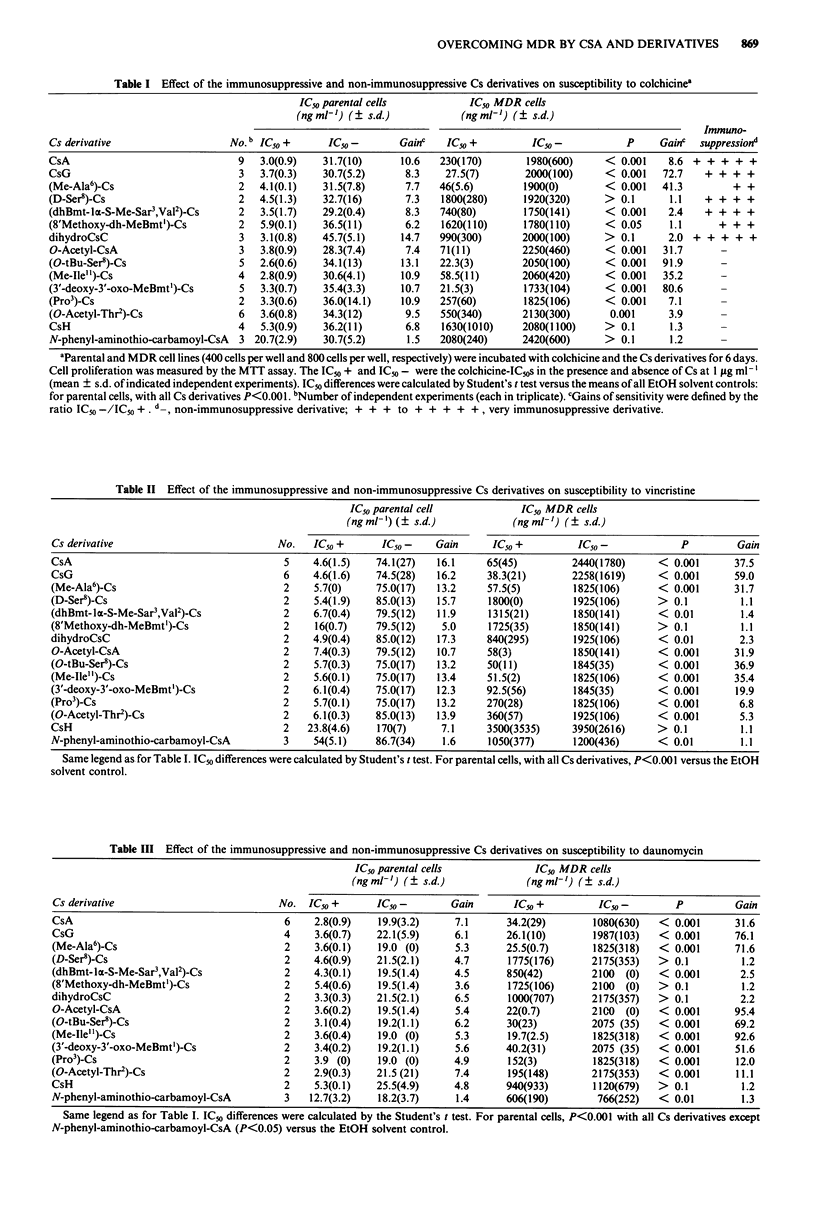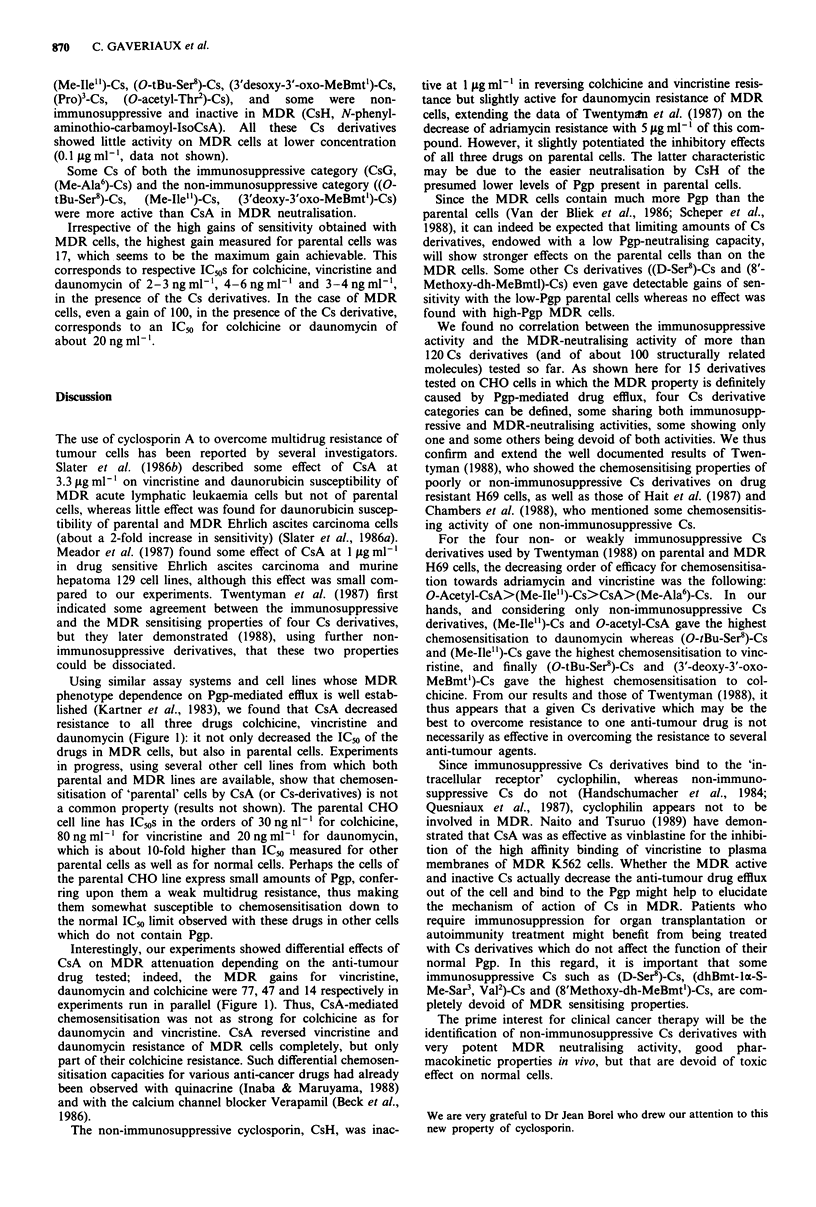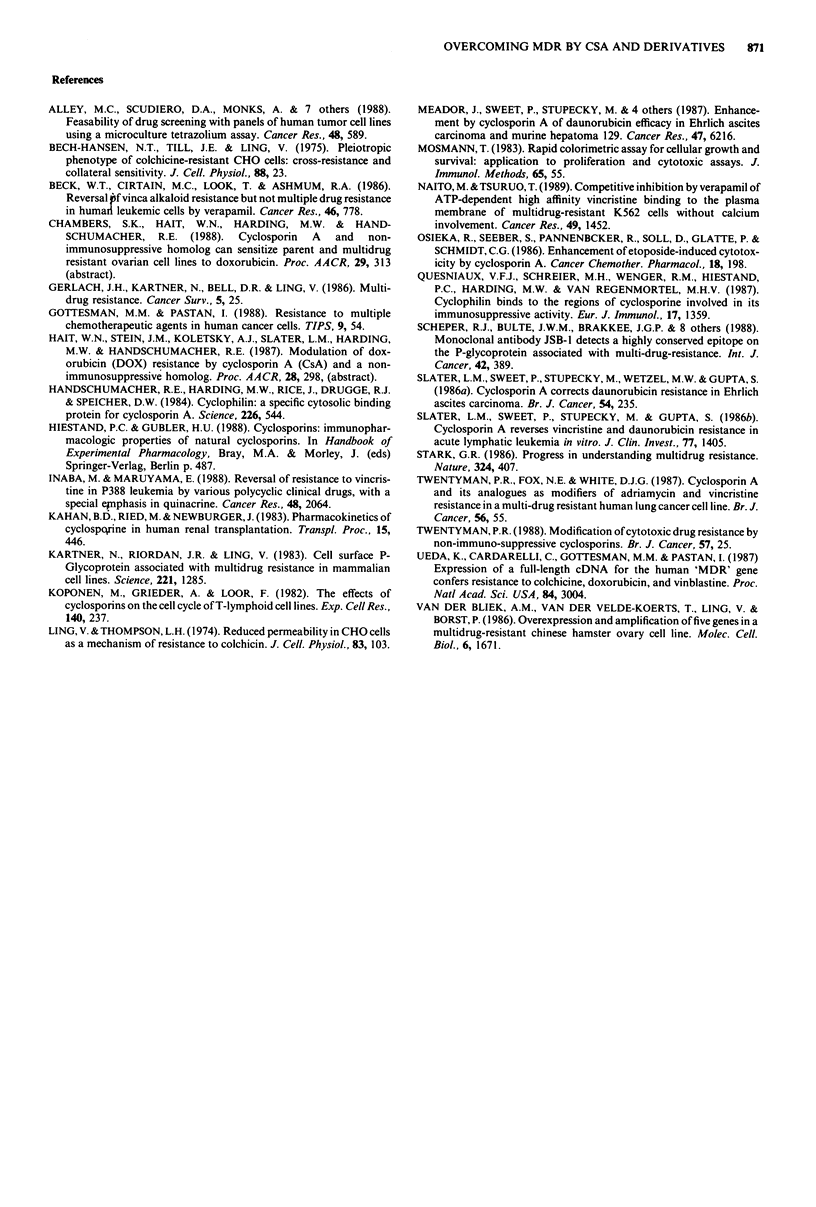# Overcoming multidrug resistance in Chinese hamster ovary cells in vitro by cyclosporin A (Sandimmune) and non-immunosuppressive derivatives.

**DOI:** 10.1038/bjc.1989.381

**Published:** 1989-12

**Authors:** C. GavÃ©riaux, D. Boesch, J. J. Boelsterli, P. Bollinger, M. K. Eberle, P. Hiestand, T. Payne, R. Traber, R. Wenger, F. Loor

**Affiliations:** Preclinical Research Department, Sandoz, CH 4002 Basel, Switzerland.

## Abstract

Cyclosporin A (Sandimmune) increased the in vitro susceptibility of 'parental' and 'multidrug-resistant' (MDR) chinese hamster ovary (CHO) cell lines to three anti-tumour drugs: colchicine, daunomycin, and vincristine. Several immunosuppressive or non-immunosuppressive derivatives of cyclosporin (Cs) were compared for their ability to sensitise both parental and MDR cells to chemotherapeutic agents. Although 5-10-fold increases of sensitivity to anti-tumour drugs could be obtained for cells of the parental line with several Cs-derivatives, the largest 'gains' of sensitivity (chemosensitisation) were obtained for the cells of the MDR line and with only some of the Cs derivatives. The MDR cells employed displayed the typical MDR phenotype. However, we found no correlation between the immunosuppressive activity of Cs derivatives and their capacity to reverse MDR and all four possible combinations of these two activities could indeed be shown among the tested Cs derivatives. This study demonstrates for the first time that some immunosuppressive Cs can be devoid of chemosensitising activity.


Br. J. Cancer (1989), 60, 867–871 © The Macmillan Press Ltd., 1989

### Overcoming multidrug resistance in Chinese hamster ovary cells *in vitro* by cyclosporin A (Sandimmune) and non-immunosuppressive derivatives

C. Gavériaux, D. Boesch, J.J. Boelsterli, P. Bollinger, M.K. Eberle, P. Hiestand, T. Payne, R. Traber, R. Wenger & F. Loor

*Preclinical Research Department 386/125, Sandoz, CH 4002 Basel, Switzerland.*

**Summary** Cyclosporin A (Sandimmune) increased the *in vitro* susceptibility of 'parental' and 'multidrug-resistant' (MDR) chinese hamster ovary (CHO) cell lines to three anti-tumour drugs: colchicine, daunomycin, and vincristine. Several immunosuppressive or non-immunosuppressive derivatives of cyclosporin (Cs) were compared for their ability to sensitise both parental and MDR cells to chemotherapeutic agents. Although 5–10-fold increases of sensitivity to anti-tumour drugs could be obtained for cells of the parental line with several Cs-derivatives, the largest 'gains' of sensitivity (chemosensitisation) were obtained for the cells of the MDR line and with only some of the Cs derivatives. The MDR cells employed displayed the typical MDR phenotype. However, we found no correlation between the immunosuppressive activity of Cs derivatives and their capacity to reverse MDR and all *four* possible combinations of these two activities could indeed be shown among the tested Cs derivatives. This study demonstrates for the first time that some immunosuppressive Cs can be devoid of chemosensitising activity.

Prolonged treatment of tumour cells with an anti-cancer drug may cause them to become resistant to a variety of drugs which differ in their mechanism of action, but share the property of entering the cells by passive diffusion through the membrane (Gerlach *et al.*, 1986; Stark, 1986; Gottesman & Pastan, 1988). This multidrug resistance (MDR) has been closely linked to the specific amplification of expression of a particular class of transmembrane glycoprotein called the P-glycoprotein (Pgp) (Ling & Thompson, 1974; Bech-Hansen *et al.*, 1975; Kartner *et al.*, 1983; Ueda *et al.*, 1987). The P-glycoproteins decrease the intracellular concentration of the anti-cancer drug below its cytostatic threshold by actively pumping it out of the cell.

Various membrane active agents (calcium antagonists, local anaesthetics) or compounds reducing intracellular ATP levels have been shown to interfere with the Pgp function. The immunosuppressive drug cyclosporin A (CsA, Sandimmune) has already been shown to reverse MDR. CsA corrects the daunorubicin resistance in Ehrlich ascites carcinoma (Slater *et al.*, 1986*a*) and the daunorubicin and vincristine resistance in acute lymphatic leukaemia (Slater *et al.*, 1986*b*). CsA also enhances the daunorubicin efficacy in murine hepatoma (Meador *et al.*, 1987), modifies adriamycin and vincristine resistance in a MDR human lung cancer cell line (Twentyman *et al.*, 1987), and enhances the cytotoxicity of etoposide and adriamycin in L1210 leukaemic cells (Osieka *et al.*, 1986).

Two properties of CsA limit its use as a resistance modifying agent in cancer chemotherapy. They are the immunosuppressive potency of the drug and its clinical side-effects, especially nephrotoxicity, neither of which would be acceptable in high dose cancer treatment.

It was thus mandatory to search for cyclosporin (Cs) analogues lacking both these unwanted effects of CsA but displaying similar or enhanced activity in sensitising MDR tumours towards anti-cancer drugs. For this purpose, we established a screening programme for Cs-derivatives using well known MDR and parental (control) cell lines, i.e. the chemotherapy resistant and sensitive Chinese hamster ovary (CHO) cell lines established by Dr V. Ling (Toronto) and used in many laboratories around the world.

Correspondence: F. Loor, Laboratoire d'Immunologie, Faculté de Pharmacie, Université Louis Pasteur Strasbourg 1, BP 24, 67401 Illkirch-cedex, France.
Received 10 March 1989; and in revised form 20 June 1989.

### Materials and methods

### *Cell lines and drugs*

Chinese hamster ovary (CHO) cells were obtained from Dr V. Ling (Ontario Cancer Research Institute, Toronto, Canada): a colchicine-resistant cell line (MDR line, $CH^RC5$) and the parental colchicine-sensitive cell line AUX B1 (Ling & Thompson, 1974, Bech-Hansen *et al.*, 1975). These cell lines were grown in culture medium (αMEM medium supplemented with Asn $0.02\ mg\ ml^{-1}$, vitamins (1 ×), penicillin-streptomycin $100\ IU\ ml^{-1}$, Gln 2 mM and 10% heat inactivated fetal calf serum (all from Gibco)). Colchicine (Sandoz), daunomycin (Sigma D-4885), puromycin (Sigma P-7255), vincristine (Serva 38215) and gramicidin D (Serva 24150) were prepared as stock solutions in culture medium.

### *Immunosuppression*

The degree of immunosuppressive activity of the different Cs derivatives had been previously assessed in several *in vitro* and *in vivo* models (Hiestand & Gubler, 1988).

### *Proliferation assay of CHO cell lines*

In preliminary experiments, we measured cell growth by methods such as $^3H$-thymidine uptake or a colorimetric assay (cell mass measurement by hexosaminidase content) (Koponen *et al.*, 1982), but another colorimetric assay using MTT (Mosmann, 1983) was found to be the most convenient for screening large numbers of derivatives. This assay had also been found very 'feasible' for drug screening with panels of tumour cell lines (Alley *et al.*, 1988).

Preliminary experiments (not shown) were performed in order to determine the culture conditions. We chose to dispense, per well, twice as many MDR cells as parental cells, so that similar cell numbers, giving optical density (OD) values in the correct range (0.8–1.4, in the colorimetric assay), were reached after a 6 day culture.

In 96-well microplates (Costar 3596), 50 μl of colchicine (or another anti-cancer drug) solution were added in culture medium in triplicate to obtain final concentrations of 0, $0.1-30\ \mu g\ ml^{-1}$ for the MDR line, and 0, $0.001-0.3\ \mu g\ ml^{-1}$ for the parental line. A further down-extension of the dose range was performed when necessary, i.e. when a Cs derivative was strongly decreasing the anti-cancer drug $IC_{50}$ (i.e. the drug dose required to reduce the final OD to 50% of control).

The Cs derivatives to be tested were dissolved at 1 mg ml$^{-1}$ in absolute ethanol (EtOH, Merck) and were tested at 1 µg ml$^{-1}$, with control being treated with the corresponding ethanol solvent dilutions. The cyclosporin derivatives or control solutions were added (50 µl) to each well, and mixed the 100 µl cell suspensions (4 × 10$^3$ cells ml$^{-1}$ for the parental line and 8 × 10$^3$ cells ml$^{-1}$ for the MDR line) and colchicine solutions (50 µl) which had been added beforehand.

After a 6-day incubation at 37°C, the final cell number was measured by a colorimetric assay using MTT (3-[4,5-dimethylthiazol-2-yl]-2,5-diphenyl tetrazolium bromide; Sigma) (Mosmann, 1983). First, 100 µl of supernatant were removed, and then to the remaining cell suspension, 10 µl of the MTT solution (5 mg ml$^{-1}$) were added per well and the plates incubated for 3 h at 37°C; 100 µl of solvent (butanol-2, isopropanol, HCl 1 N in volume ratio 16/8/1) were added per well and the plates shaken until complete dissolution of the formazan crystals. The OD was read at 540 nm on a plate reader (Titertek Multiskan). The extent of cell growth was represented as a function of the anti-cancer drug concentration (the growth in the absence of anti-cancer drug (Cs or solvent alone) being taken as 100%).

*Data analysis*

The anti-cancer drug IC$_{50}$s were determined from the concentration–response curves: either in the presence of Cs (IC$_{50}$ + ), or in its absence (IC$_{50}$ − ) (but in the presence of the Cs solvent, i.e. ethanol).

The increases of anti-cancer drug sensitivity or 'gain of sensitivity' brought by each Cs at 1 µg ml$^{-1}$ were given by the ratio IC$_{50}$ − /IC$_{50}$ +. These measurements were performed for both cell lines (parental and MDR), for the various anti-cancer drugs assayed, and for 1 µg ml$^{-1}$ Cs.

### Results

In our assay system, we studied the effects of CsA and some of its derivatives at a concentration of 1 µg ml$^{-1}$. Indeed, maximum tolerable plasma levels of CsA are in the order of 1–2 µg ml$^{-1}$ (Kahan *et al.*, 1983). Moreover, none of the Cs derivatives reported in this paper was toxic by itself at 1 µg ml$^{-1}$ on parental or MDR CHO cells, no detectable effect on their growth being detected up to 3 µg ml$^{-1}$ for all of them, and up to 10 µg ml$^{-1}$ for most of them.

*Effect of CsA, CsH and N-phenylaminothio-carbamoyl-CsA in combination with the anti-tumour drugs on the growth of parental and MDR lines*

The parental and MDR cell lines were first compared for sensitivity to colchicine, daunomycin and vincristine, and the IC$_{50}$ (as µg ml$^{-1}$) was determined for each drug. Colchicine, daunomycin and vincristine inhibited the cell growth at comparable concentrations (Figure 1, open and filled circles, for parental and MDR cells respectively). Gramicidin D and puromycin required high doses to inhibit the MDR line (IC$_{50}$s>50 µg ml$^{-1}$, not shown). Figure 1 shows the effect of CsA, CsH and *N*-phenylaminothio-carbamoyl-CsA on the drug resistance of both CHO cell lines (the control growth in the absence of anti-tumour drug being considered as 100%), the dose–response curves being established with colchicine, vincristine and daunomycin. For the potentation of all three anti-cancer drugs (gains of sensitivity), CsA was the most active: the vincristine IC$_{50}$ and daunomycin IC$_{50}$ of MDR cells in the presence of CsA even fell below the vincristine IC$_{50}$ and daunomycin IC$_{50}$ of parental line in the absence of CsA. CsA was also effective on the parental line itself, the parental gains and MDR gains being respectively 10 and 14 for colchicine, 11 and 47 for daunomycin, and 22 and 77 for vincristine. The *N*-phenylaminothio-carbamoyl-CsA was essentially inactive, the gains of sensitivity being below 2. CsH, an immunologically inactive derivative of CsA, was weakly active in our assay, giving parental gains and MDR gains of, respectively, 4 and 2.5 for daunomycin, 4.5 and 2 for vincristine, and 6 and 1.2 for colchicine.

**Figure 1** Effect of CsA, CsH and *N*-phenyl-aminothio-carbamoyl-CsA on multidrug-resistance. Parental (○ □ △ ▽) and MDR (● ■ ▲ ▼) cell lines were cultured with colchicine (**a**), vincristine (**b**) and daunomycin (**c**) for 6 days in the presence of CsA (△ ▲), CsH (□ ■), *N*-phenyl-aminothio-carbamoyl-CsA (▽ ▼) at 1 µg ml$^{-1}$, or the ethanol solvent control (○ ●). Cell proliferation is represented as percentage of the control cell growth, that is growth without Cs and without anti-tumour drugs, versus anti-tumour drug concentration in µg ml$^{-1}$ (mean of four independent experiments in triplicate).

*Effect of some Cs derivatives on resistance to colchicine, vincristine and daunomycin*

The IC$_{50}$ ranges for colchicine, vincristine and daunomycin in our assay were the following, respectively (in ng ml$^{-1}$): 18.5–53, 50–175 and 14–23.5 for parental line, and 980–3,100, 660–5,900 and 500–2,100 for the MDR line.

The ability of some Cs derivatives with different immunosuppressive powers to sensitise parental and MDR CHO cells to the three anti-tumour drugs is shown in Table I for colchicine, in Table II for vincristine and in Table III for daunomycin. There is a good agreement between the effects of these 15 Cs derivatives on colchicine, vincristine and daunomycin resistance. Some Cs derivatives could overcome colchicine, vincristine and daunomycin resistance, thus giving an IC$_{50}$ + for MDR cells similar to the IC$_{50}$ of parental cells: CsA, CsG, (Me-Ala$^6$)-Cs, *O*-Acetyl-CsA, (*O*-tBu-Ser$^8$)-Cs, (Me-Ile$^{11}$)-Cs and (3′deoxy-3′-oxo-MeBmt$^1$)-Cs.

No correlation was found between the immunosuppressive and MDR sensitising properties of these 15 derivatives. Some Cs were both immunosuppressive and active in MDR (CsA, CsG, (Me-Ala$^6$)-Cs), some were immunosuppressive but inactive in MDR ((D-Ser$^8$)Cs, (dhBmt-1,α-S-Me-Sar$^3$,Val$^2$)-Cs, (8′Methoxy-dh-MeBmt$^1$)Cs, dihydroCsC), some were non-immunosuppressive but active in MDR (*O*-acetyl-CsA,

**Table I** Effect of the immunosuppressive and non-immunosuppressive Cs derivatives on susceptibility to colchicine[a]

| Cs derivative | No.[b] | $IC_{50}$ parental cells ($ng\ ml^{-1}$) (± s.d.) $IC_{50}$ + | $IC_{50}$ − | Gain[c] | $IC_{50}$ MDR cells ($ng\ ml^{-1}$) (± s.d.) $IC_{50}$ + | $IC_{50}$ − | P | Gain[c] | Immuno-suppression[d] |
|---|---|---|---|---|---|---|---|---|---|
| CsA | 9 | 3.0(0.9) | 31.7(10) | 10.6 | 230(170) | 1980(600) | < 0.001 | 8.6 | + + + + + |
| CsG | 3 | 3.7(0.3) | 30.7(5.2) | 8.3 | 27.5(7) | 2000(100) | < 0.001 | 72.7 | + + + + |
| (Me-Ala[6])-Cs | 2 | 4.1(0.1) | 31.5(7.8) | 7.7 | 46(5.6) | 1900(0) | < 0.001 | 41.3 | + + |
| (D-Ser[8])-Cs | 2 | 4.5(1.3) | 32.7(16) | 7.3 | 1800(280) | 1920(320) | > 0.1 | 1.1 | + + + + |
| (dhBmt-1α-S-Me-Sar[3],Val[2])-Cs | 2 | 3.5(1.7) | 29.2(0.4) | 8.3 | 740(80) | 1750(141) | < 0.001 | 2.4 | + + + + |
| (8'Methoxy-dh-MeBmt[1])-Cs | 2 | 5.9(0.1) | 36.5(11) | 6.2 | 1620(110) | 1780(110) | < 0.05 | 1.1 | + + + |
| dihydroCsC | 3 | 3.1(0.8) | 45.7(5.1) | 14.7 | 990(300) | 2000(100) | > 0.1 | 2.0 | + + + + + |
| *O*-Acetyl-CsA | 3 | 3.8(0.9) | 28.3(7.4) | 7.4 | 71(11) | 2250(460) | < 0.001 | 31.7 | − |
| (*O*-tBu-Ser[8])-Cs | 5 | 2.6(0.6) | 34.1(13) | 13.1 | 22.3(3) | 2050(100) | < 0.001 | 91.9 | − |
| (Me-Ile[11])-Cs | 4 | 2.8(0.9) | 30.6(4.1) | 10.9 | 58.5(11) | 2060(420) | < 0.001 | 35.2 | − |
| (3'-deoxy-3'-oxo-MeBmt[1])-Cs | 5 | 3.3(0.7) | 35.4(3.3) | 10.7 | 21.5(3) | 1733(104) | < 0.001 | 80.6 | − |
| (Pro[3])-Cs | 2 | 3.3(0.6) | 36.0(14.1) | 10.9 | 257(60) | 1825(106) | < 0.001 | 7.1 | − |
| (*O*-Acetyl-Thr[2])-Cs | 6 | 3.6(0.8) | 34.3(12) | 9.5 | 550(340) | 2130(300) | 0.001 | 3.9 | − |
| CsH | 4 | 5.3(0.9) | 36.2(11) | 6.8 | 1630(1010) | 2080(1100) | > 0.1 | 1.3 | − |
| *N*-phenyl-aminothio-carbamoyl-CsA | 3 | 20.7(2.9) | 30.7(5.2) | 1.5 | 2080(240) | 2420(600) | > 0.1 | 1.2 | − |

[a]Parental and MDR cell lines (400 cells per well and 800 cells per well, respectively) were incubated with colchicine and the Cs derivatives for 6 days. Cell proliferation was measured by the MTT assay. The $IC_{50}$ + and $IC_{50}$ − were the colchicine-$IC_{50}$s in the presence and absence of Cs at 1 $\mu g\ ml^{-1}$ (mean ± s.d. of indicated independent experiments). $IC_{50}$ differences were calculated by Student's *t* test versus the means of all EtOH solvent controls: for parental cells, with all Cs derivatives $P<0.001$. [b]Number of independent experiments (each in triplicate). [c]Gains of sensitivity were defined by the ratio $IC_{50}$ −/$IC_{50}$ +. [d]−, non-immunosuppressive derivative; + + + to + + + + +, very immunosuppressive derivative.

**Table II** Effect of the immunosuppressive and non-immunosuppressive Cs derivatives on susceptibility to vincristine

| Cs derivative | No. | $IC_{50}$ parental cell ($ng\ ml^{-1}$) (± s.d.) $IC_{50}$ + | $IC_{50}$ − | Gain | $IC_{50}$ MDR cells ($ng\ ml^{-1}$) (± s.d.) $IC_{50}$ + | $IC_{50}$ − | P | Gain |
|---|---|---|---|---|---|---|---|---|
| CsA | 5 | 4.6(1.5) | 74.1(27) | 16.1 | 65(45) | 2440(1780) | < 0.001 | 37.5 |
| CsG | 6 | 4.6(1.6) | 74.5(28) | 16.2 | 38.3(21) | 2258(1619) | < 0.001 | 59.0 |
| (Me-Ala[6])-Cs | 2 | 5.7(0) | 75.0(17) | 13.2 | 57.5(5) | 1825(106) | < 0.001 | 31.7 |
| (D-Ser[8])-Cs | 2 | 5.4(1.9) | 85.0(13) | 15.7 | 1800(0) | 1925(106) | > 0.1 | 1.1 |
| (dhBmt-1α-S-Me-Sar[3],Val[2])-Cs | 2 | 6.7(0.4) | 79.5(12) | 11.9 | 1315(21) | 1850(141) | < 0.01 | 1.4 |
| (8'Methoxy-dh-MeBmt[1])-Cs | 2 | 16(0.7) | 79.5(12) | 5.0 | 1725(35) | 1850(141) | > 0.1 | 1.1 |
| dihydroCsC | 2 | 4.9(0.4) | 85.0(12) | 17.3 | 840(295) | 1925(106) | < 0.01 | 2.3 |
| *O*-Acetyl-CsA | 2 | 7.4(0.3) | 79.5(12) | 10.7 | 58(3) | 1850(141) | < 0.001 | 31.9 |
| (*O*-tBu-Ser[8])-Cs | 2 | 5.7(0.3) | 75.0(17) | 13.2 | 50(11) | 1845(35) | < 0.001 | 36.9 |
| (Me-Ile[11])-Cs | 2 | 5.6(0.1) | 75.0(17) | 13.4 | 51.5(2) | 1825(106) | < 0.001 | 35.4 |
| (3'-deoxy-3'-oxo-MeBmt[1])-Cs | 2 | 6.1(0.4) | 75.0(17) | 12.3 | 92.5(56) | 1845(35) | < 0.001 | 19.9 |
| (Pro[3])-Cs | 2 | 5.7(0.1) | 75.0(17) | 13.2 | 270(28) | 1825(106) | < 0.001 | 6.8 |
| (*O*-Acetyl-Thr[2])-Cs | 2 | 6.1(0.3) | 85.0(13) | 13.9 | 360(57) | 1925(106) | < 0.001 | 5.3 |
| CsH | 2 | 23.8(4.6) | 170(7) | 7.1 | 3500(3535) | 3950(2616) | > 0.1 | 1.1 |
| *N*-phenyl-aminothio-carbamoyl-CsA | 3 | 54(5.1) | 86.7(34) | 1.6 | 1050(377) | 1200(436) | < 0.01 | 1.1 |

Same legend as for Table I. $IC_{50}$ differences were calculated by Student's *t* test. For parental cells, with all Cs derivatives, $P<0.001$ versus the EtOH solvent control.

**Table III** Effect of the immunosuppressive and non-immunosuppressive Cs derivatives on susceptibility to daunomycin

| Cs derivative | No. | $IC_{50}$ parental cells ($ng\ ml^{-1}$) (± s.d.) $IC_{50}$ + | $IC_{50}$ − | Gain | $IC_{50}$ MDR cells ($ng\ ml^{-1}$) (± s.d.) $IC_{50}$ + | $IC_{50}$ − | P | Gain |
|---|---|---|---|---|---|---|---|---|
| CsA | 6 | 2.8(0.9) | 19.9(3.2) | 7.1 | 34.2(29) | 1080(630) | < 0.001 | 31.6 |
| CsG | 4 | 3.6(0.7) | 22.1(5.9) | 6.1 | 26.1(10) | 1987(103) | < 0.001 | 76.1 |
| (Me-Ala[6])-Cs | 2 | 3.6(0.1) | 19.0 (0) | 5.3 | 25.5(0.7) | 1825(318) | < 0.001 | 71.6 |
| (*D*-Ser[8])-Cs | 2 | 4.6(0.9) | 21.5(2.1) | 4.7 | 1775(176) | 2175(353) | > 0.1 | 1.2 |
| (dhBmt-1α-S-Me-Sar[3],Val[2])-Cs | 2 | 4.3(0.1) | 19.5(1.4) | 4.5 | 850(42) | 2100 (0) | < 0.001 | 2.5 |
| (8'Methoxy-dh-MeBmt[1])-Cs | 2 | 5.4(0.6) | 19.5(1.4) | 3.6 | 1725(106) | 2100 (0) | > 0.1 | 1.2 |
| dihydroCsC | 2 | 3.3(0.3) | 21.5(2.1) | 6.5 | 1000(707) | 2175(357) | > 0.1 | 2.2 |
| *O*-Acetyl-CsA | 2 | 3.6(0.2) | 19.5(1.4) | 5.4 | 22(0.7) | 2100 (0) | < 0.001 | 95.4 |
| (*O*-tBu-Ser[8])-Cs | 2 | 3.1(0.4) | 19.2(1.1) | 6.2 | 30(23) | 2075 (35) | < 0.001 | 69.2 |
| (Me-Ile[11])-Cs | 2 | 3.6(0.4) | 19.0 (0) | 5.3 | 19.7(2.5) | 1825(318) | < 0.001 | 92.6 |
| (3'-deoxy-3'-oxo-MeBmt[1])-Cs | 2 | 3.4(0.2) | 19.2(1.1) | 5.6 | 40.2(31) | 2075 (35) | < 0.001 | 51.6 |
| (Pro[3])-Cs | 2 | 3.9 (0) | 19.0 (0) | 4.9 | 152(3) | 1825(318) | < 0.001 | 12.0 |
| (*O*-Acetyl-Thr[2])-Cs | 2 | 2.9(0.3) | 21.5 (21) | 7.4 | 195(148) | 2175(353) | < 0.001 | 11.1 |
| CsH | 2 | 5.3(0.1) | 25.5(4.9) | 4.8 | 940(933) | 1120(679) | > 0.1 | 1.2 |
| *N*-phenyl-aminothio-carbamoyl-CsA | 3 | 12.7(3.2) | 18.2(3.7) | 1.4 | 606(190) | 766(252) | < 0.01 | 1.3 |

Same legend as for Table I. $IC_{50}$ differences were calculated by the Student's *t* test. For parental cells, $P<0.001$ with all Cs derivatives except *N*-phenyl-aminothio-carbamoyl-CsA ($P<0.05$) versus the EtOH solvent control.

(Me-Ile[11])-Cs, (O-tBu-Ser[8])-Cs, (3'desoxy-3'-oxo-MeBmt[1])-Cs, (Pro)[3]-Cs, (O-acetyl-Thr[2])-Cs), and some were non-immunosuppressive and inactive in MDR (CsH, N-phenyl-aminothio-carbamoyl-IsoCsA). All these Cs derivatives showed little activity on MDR cells at lower concentration (0.1 $\mu g\ ml^{-1}$, data not shown).

Some Cs of both the immunosuppressive category (CsG, (Me-Ala[6])-Cs) and the non-immunosuppressive category ((O-tBu-Ser[8])-Cs, (Me-Ile[11])-Cs, (3'deoxy-3'oxo-MeBmt[1])-Cs) were more active than CsA in MDR neutralisation.

Irrespective of the high gains of sensitivity obtained with MDR cells, the highest gain measured for parental cells was 17, which seems to be the maximum gain achievable. This corresponds to respective $IC_{50}$s for colchicine, vincristine and daunomycin of 2–3 $ng\ ml^{-1}$, 4–6 $ng\ ml^{-1}$ and 3–4 $ng\ ml^{-1}$, in the presence of the Cs derivatives. In the case of MDR cells, even a gain of 100, in the presence of the Cs derivative, corresponds to an $IC_{50}$ for colchicine or daunomycin of about 20 $ng\ ml^{-1}$.

### Discussion

The use of cyclosporin A to overcome multidrug resistance of tumour cells has been reported by several investigators. Slater et al. (1986b) described some effect of CsA at 3.3 $\mu g\ ml^{-1}$ on vincristine and daunorubicin susceptibility of MDR acute lymphatic leukaemia cells but not of parental cells, whereas little effect was found for daunorubicin susceptibility of parental and MDR Ehrlich ascites carcinoma cells (about a 2-fold increase in sensitivity) (Slater et al., 1986a). Meador et al. (1987) found some effect of CsA at 1 $\mu g\ ml^{-1}$ in drug sensitive Ehrlich ascites carcinoma and murine hepatoma 129 cell lines, although this effect was small compared to our experiments. Twentyman et al. (1987) first indicated some agreement between the immunosuppressive and the MDR sensitising properties of four Cs derivatives, but they later demonstrated (1988), using further non-immunosuppressive derivatives, that these two properties could be dissociated.

Using similar assay systems and cell lines whose MDR phenotype dependence on Pgp-mediated efflux is well established (Kartner et al., 1983), we found that CsA decreased resistance to all three drugs colchicine, vincristine and daunomycin (Figure 1): it not only decreased the $IC_{50}$ of the drugs in MDR cells, but also in parental cells. Experiments in progress, using several other cell lines from which both parental and MDR lines are available, show that chemosensitisation of 'parental' cells by CsA (or Cs-derivatives) is not a common property (results not shown). The parental CHO cell line has $IC_{50}$s in the orders of 30 $ng\ nl^{-1}$ for colchicine, 80 $ng\ ml^{-1}$ for vincristine and 20 $ng\ ml^{-1}$ for daunomycin, which is about 10-fold higher than $IC_{50}$ measured for other parental cells as well as for normal cells. Perhaps the cells of the parental CHO line express small amounts of Pgp, conferring upon them a weak multidrug resistance, thus making them somewhat susceptible to chemosensitisation down to the normal $IC_{50}$ limit observed with these drugs in other cells which do not contain Pgp.

Interestingly, our experiments showed differential effects of CsA on MDR attenuation depending on the anti-tumour drug tested; indeed, the MDR gains for vincristine, daunomycin and colchicine were 77, 47 and 14 respectively in experiments run in parallel (Figure 1). Thus, CsA-mediated chemosensitisation was not as strong for colchicine as for daunomycin and vincristine. CsA reversed vincristine and daunomycin resistance of MDR cells completely, but only part of their colchicine resistance. Such differential chemosensitisation capacities for various anti-cancer drugs had already been observed with quinacrine (Inaba & Maruyama, 1988) and with the calcium channel blocker Verapamil (Beck et al., 1986).

The non-immunosuppressive cyclosporin, CsH, was inactive at 1 $\mu g\ ml^{-1}$ in reversing colchicine and vincristine resistance but slightly active for daunomycin resistance of MDR cells, extending the data of Twentyman et al. (1987) on the decrease of adriamycin resistance with 5 $\mu g\ ml^{-1}$ of this compound. However, it slightly potentiated the inhibitory effects of all three drugs on parental cells. The latter characteristic may be due to the easier neutralisation by CsH of the presumed lower levels of Pgp present in parental cells.

Since the MDR cells contain much more Pgp than the parental cells (Van der Bliek et al., 1986; Scheper et al., 1988), it can indeed be expected that limiting amounts of Cs derivatives, endowed with a low Pgp-neutralising capacity, will show stronger effects on the parental cells than on the MDR cells. Some other Cs derivatives ((D-Ser[8])-Cs and (8'-Methoxy-dh-MeBmtl)-Cs) even gave detectable gains of sensitivity with the low-Pgp parental cells whereas no effect was found with high-Pgp MDR cells.

We found no correlation between the immunosuppressive activity and the MDR-neutralising activity of more than 120 Cs derivatives (and of about 100 structurally related molecules) tested so far. As shown here for 15 derivatives tested on CHO cells in which the MDR property is definitely caused by Pgp-mediated drug efflux, four Cs derivative categories can be defined, some sharing both immunosuppressive and MDR-neutralising activities, some showing only one and some others being devoid of both activities. We thus confirm and extend the well documented results of Twentyman (1988), who showed the chemosensitising properties of poorly or non-immunosuppressive Cs derivatives on drug resistant H69 cells, as well as those of Hait et al. (1987) and Chambers et al. (1988), who mentioned some chemosensitising activity of one non-immunosuppressive Cs.

For the four non- or weakly immunosuppressive Cs derivatives used by Twentyman (1988) on parental and MDR H69 cells, the decreasing order of efficacy for chemosensitisation towards adriamycin and vincristine was the following: O-Acetyl-CsA>(Me-Ile[11])-Cs>CsA>(Me-Ala[6])-Cs. In our hands, and considering only non-immunosuppressive Cs derivatives, (Me-Ile[11])-Cs and O-acetyl-CsA gave the highest chemosensitisation to daunomycin whereas (O-tBu-Ser[8])-Cs and (Me-Ile[11])-Cs gave the highest chemosensitisation to vincristine, and finally (O-tBu-Ser[8])-Cs and (3'-deoxy-3'-oxo-MeBmt[1])-Cs gave the highest chemosensitisation to colchicine. From our results and those of Twentyman (1988), it thus appears that a given Cs derivative which may be the best to overcome resistance to one anti-tumour drug is not necessarily as effective in overcoming the resistance to several anti-tumour agents.

Since immunosuppressive Cs derivatives bind to the 'intracellular receptor' cyclophilin, whereas non-immunosuppressive Cs do not (Handschumacher et al., 1984; Quesniaux et al., 1987), cyclophilin appears not to be involved in MDR. Naito and Tsuruo (1989) have demonstrated that CsA was as effective as vinblastine for the inhibition of the high affinity binding of vincristine to plasma membranes of MDR K562 cells. Whether the MDR active and inactive Cs actually decrease the anti-tumour drug efflux out of the cell and bind to the Pgp might help to elucidate the mechanism of action of Cs in MDR. Patients who require immunosuppression for organ transplantation or autoimmunity treatment might benefit from being treated with Cs derivatives which do not affect the function of their normal Pgp. In this regard, it is important that some immunosuppressive Cs such as (D-Ser[8])-Cs, (dhBmt-1α-S-Me-Sar[3], Val[2])-Cs and (8'Methoxy-dh-MeBmt[1])-Cs, are completely devoid of MDR sensitising properties.

The prime interest for clinical cancer therapy will be the identification of non-immunosuppressive Cs derivatives with very potent MDR neutralising activity, good pharmacokinetic properties *in vivo*, but that are devoid of toxic effect on normal cells.

We are very grateful to Dr Jean Borel who drew our attention to this new property of cyclosporin.

### References

ALLEY, M.C., SCUDIERO, D.A., MONKS, A. & 7 others (1988). Feasability of drug screening with panels of human tumor cell lines using a microculture tetrazolium assay. *Cancer Res.*, **48**, 589.

BECH-HANSEN, N.T., TILL, J.E. & LING, V. (1975). Pleiotropic phenotype of colchicine-resistant CHO cells: cross-resistance and collateral sensitivity. *J. Cell. Physiol.*, **88**, 23.

BECK, W.T., CIRTAIN, M.C., LOOK, T. & ASHMUM, R.A. (1986). Reversal of vinca alkaloid resistance but not multiple drug resistance in human leukemic cells by verapamil. *Cancer Res.*, **46**, 778.

CHAMBERS, S.K., HAIT, W.N., HARDING, M.W. & HANDSCHUMACHER, R.E. (1988). Cyclosporin A and non-immunosuppressive homolog can sensitize parent and multidrug resistant ovarian cell lines to doxorubicin. *Proc. AACR*, **29**, 313 (abstract).

GERLACH, J.H., KARTNER, N., BELL, D.R. & LING, V. (1986). Multidrug resistance. *Cancer Surv.*, **5**, 25.

GOTTESMAN, M.M. & PASTAN, I. (1988). Resistance to multiple chemotherapeutic agents in human cancer cells. *TIPS*, **9**, 54.

HAIT, W.N., STEIN, J.M., KOLETSKY, A.J., SLATER, L.M., HARDING, M.W. & HANDSCHUMACHER, R.E. (1987). Modulation of doxorubicin (DOX) resistance by cyclosporin A (CsA) and a non-immunosuppressive homolog. *Proc. AACR*, **28**, 298, (abstract).

HANDSCHUMACHER, R.E., HARDING, M.W., RICE, J., DRUGGE, R.J. & SPEICHER, D.W. (1984). Cyclophilin: a specific cytosolic binding protein for cyclosporin A. *Science*, **226**, 544.

HIESTAND, P.C. & GUBLER, H.U. (1988). Cyclosporins: immunopharmacologic properties of natural cyclosporins. In *Handbook of Experimental Pharmacology*, Bray, M.A. & Morley, J. (eds) Springer-Verlag, Berlin p. 487.

INABA, M. & MARUYAMA, E. (1988). Reversal of resistance to vincristine in P388 leukemia by various polycyclic clinical drugs, with a special emphasis in quinacrine. *Cancer Res.*, **48**, 2064.

KAHAN, B.D., RIED, M. & NEWBURGER, J. (1983). Pharmacokinetics of cyclosporine in human renal transplantation. *Transpl. Proc.*, **15**, 446.

KARTNER, N., RIORDAN, J.R. & LING, V. (1983). Cell surface P-Glycoprotein associated with multidrug resistance in mammalian cell lines. *Science*, **221**, 1285.

KOPONEN, M., GRIEDER, A. & LOOR, F. (1982). The effects of cyclosporins on the cell cycle of T-lymphoid cell lines. *Exp. Cell Res.*, **140**, 237.

LING, V. & THOMPSON, L.H. (1974). Reduced permeability in CHO cells as a mechanism of resistance to colchicin. *J. Cell. Physiol.*, **83**, 103.

MEADOR, J., SWEET, P., STUPECKY, M. & 4 others (1987). Enhancement by cyclosporin A of daunorubicin efficacy in Ehrlich ascites carcinoma and murine hepatoma 129. *Cancer Res.*, **47**, 6216.

MOSMANN, T. (1983). Rapid colorimetric assay for cellular growth and survival: application to proliferation and cytotoxic assays. *J. Immunol. Methods*, **65**, 55.

NAITO, M. & TSURUO, T. (1989). Competitive inhibition by verapamil of ATP-dependent high affinity vincristine binding to the plasma membrane of multidrug-resistant K562 cells without calcium involvement. *Cancer Res.*, **49**, 1452.

OSIEKA, R., SEEBER, S., PANNENBCKER, R., SOLL, D., GLATTE, P. & SCHMIDT, C.G. (1986). Enhancement of etoposide-induced cytotoxicity by cyclosporin A. *Cancer Chemother. Pharmacol.*, **18**, 198.

QUESNIAUX, V.F.J., SCHREIER, M.H., WENGER, R.M., HIESTAND, P.C., HARDING, M.W. & VAN REGENMORTEL, M.H.V. (1987). Cyclophilin binds to the regions of cyclosporine involved in its immunosuppressive activity. *Eur. J. Immunol.*, **17**, 1359.

SCHEPER, R.J., BULTE, J.W.M., BRAKKEE, J.G.P. & 8 others (1988). Monoclonal antibody JSB-1 detects a highly conserved epitope on the P-glycoprotein associated with multi-drug-resistance. *Int. J. Cancer*, **42**, 389.

SLATER, L.M., SWEET, P., STUPECKY, M., WETZEL, M.W. & GUPTA, S. (1986*a*). Cyclosporin A corrects daunorubicin resistance in Ehrlich ascites carcinoma. *Br. J. Cancer*, **54**, 235.

SLATER, L.M., SWEET, P., STUPECKY, M. & GUPTA, S. (1986*b*). Cyclosporin A reverses vincristine and daunorubicin resistance in acute lymphatic leukemia *in vitro*. *J. Clin. Invest.*, **77**, 1405.

STARK, G.R. (1986). Progress in understanding multidrug resistance. *Nature*, **324**, 407.

TWENTYMAN, P.R., FOX, N.E. & WHITE, D.J.G. (1987). Cyclosporin A and its analogues as modifiers of adriamycin and vincristine resistance in a multi-drug resistant human lung cancer cell line. *Br. J. Cancer*, **56**, 55.

TWENTYMAN, P.R. (1988). Modification of cytotoxic drug resistance by non-immuno-suppressive cyclosporins. *Br. J. Cancer*, **57**, 25.

UEDA, K., CARDARELLI, C., GOTTESMAN, M.M. & PASTAN, I. (1987) Expression of a full-length cDNA for the human 'MDR' gene confers resistance to colchicine, doxorubicin, and vinblastine. *Proc. Natl Acad. Sci. USA*, **84**, 3004.

VAN DER BLIEK, A.M., VAN DER VELDE-KOERTS, T., LING, V. & BORST, P. (1986). Overexpression and amplification of five genes in a multidrug-resistant chinese hamster ovary cell line. *Molec. Cell. Biol.*, **6**, 1671.